# Access to non-pecuniary benefits: does gender matter? Evidence from six low- and middle-income countries

**DOI:** 10.1186/1478-4491-9-25

**Published:** 2011-10-19

**Authors:** Neeru Gupta, Marco Alfano

**Affiliations:** 1 Health Workforce Information and Governance, World Health Organization, Geneva, Switzerland; 2 University of Warwick, Coventry, United Kingdom of Great Britain and Northern Ireland

## Abstract

**Background:**

Gender issues remain a neglected area in most approaches to health workforce policy, planning and research. There is an accumulating body of evidence on gender differences in health workers' employment patterns and pay, but inequalities in access to non-pecuniary benefits between men and women have received little attention. This study investigates empirically whether gender differences can be observed in health workers' access to non-pecuniary benefits across six low- and middle-income countries.

**Methods:**

The analysis draws on cross-nationally comparable data from health facility surveys conducted in Chad, Côte d'Ivoire, Jamaica, Mozambique, Sri Lanka and Zimbabwe. Probit regression models are used to investigate whether female and male physicians, nurses and midwives enjoy the same access to housing allowance, paid vacations, in-service training and other benefits, controlling for other individual and facility-level characteristics.

**Results:**

While the analysis did not uncover any consistent pattern of gender imbalance in access to non-monetary benefits, some important differences were revealed. Notably, female nursing and midwifery personnel (the majority of the sample) are found significantly less likely than their male counterparts to have accessed in-service training, identified not only as an incentive to attract and retain workers but also essential for strengthening workforce quality.

**Conclusion:**

This study sought to mainstream gender considerations by exploring and documenting sex differences in selected employment indicators across health labour markets. Strengthening the global evidence base about the extent to which gender is independently associated with health workforce performance requires improved generation and dissemination of sex-disaggregated data and research with particular attention to gender dimensions.

## Background

The importance of an available, competent and motivated health workforce is increasingly recognized for countries to meet their health systems objectives and achieve improved population health outcomes. In many contexts, women comprise the strong majority, often over 75%, of the health workforce [1,2]. At the same time, most health systems worldwide continue to experience occupational clustering by sex, with higher skilled medical personnel usually dominated by men, while nursing, midwifery and other 'caring' cadres are typically over-represented by women [3]. Yet gender issues remain a neglected area in most approaches to human resources for health (HRH) policy, planning and management [4]. The evidence base to support policy options for greater gender equality and improved overall productivity of the health labour force remains weak, especially in low- and middle-income countries.

Extensive research and analysis from a variety of disciplines have examined the extent to which different payment schemes (e.g. salaries, bonuses and pensions) make employees more productive. Within countries and health facilities, different types of incentives have been used to bolster staff productivity and retention, including financial as well as non-financial incentives. The latter may include: (i) incentives to address social needs of health workers, such as housing, meals, clothing, transport and childcare facilities; (ii) those to improve working conditions by, for example, offering better facilities, healthcare and personal security for workers; and (iii) professional and career path-related incentives, such as recognition schemes and opportunities for higher training and research [5-7]. Although there is an accumulating body of evidence on gender differences in health workers' employment patterns and pay (see for example [8-10]), the topic of inequalities in access to non-pecuniary incentives between men and women has received considerably less attention.

Gender mainstreaming in HRH research, policy and planning entails developing appropriate methodologies for data collection, monitoring and evaluation [1,4]. A starting point is the development or strengthening of HRH information systems that enable sex-disaggregated analysis. Health facility assessments can be a valuable component of a comprehensive HRH information system; however many previous facility-based assessments have tended to be gender blind when it comes to monitoring the staffing situation [11]. Gender analysis of the health workforce may reveal that health systems themselves can reflect or even exacerbate many of the social inequalities they are meant to address and be immune from [3]. For example, previous analysis using facility data from the Assessment of Human Resources for Health in Sri Lanka revealed potentially unintended gender imbalances in national health professional practice regulations: wide differences between men and women in rates of dual employment related to occupational differences in the right of private practice after duty hours at a government job. This is authorized for the (largely male) physician workforce but not for nurses (predominantly female) [12].

The main objective of this paper is to investigate empirically whether gender differences can be observed in health workers' access to non-pecuniary benefits, drawing on data from health facility surveys conducted in six low- and middle-income countries. The selection of countries for inclusion in the analysis is based on the nature of the information source (availability of cross-nationally comparable, sex-disaggregated data) rather than necessarily any a priori assumption of a problem of gender inequality. The policy implications of potential gender-based imbalances affecting health workforce performance and retention are also discussed.

## Methods

Our study employs data from the Assessment of Human Resources for Health, a multi-country survey implemented with technical and financial support from the World Health Organization between 2002 and 2004 in Chad, Côte d'Ivoire, Jamaica, Mozambique, Sri Lanka and Zimbabwe [12,13]. The Assessment used standardized guidelines for survey sampling (stratified random samples of health facilities and staff), data collection (model questionnaires) and data processing (model data entry and management software templates) to enhance comparability of results across countries. Data were collected via personal interviews with facility-based health service providers on a number of topics, including professional qualifications, demographic characteristics, working conditions, and financial and non-financial incentives. In particular, the survey instrument allowed health worker indicators to be disaggregated by sex.

General findings from the surveys, including analysis of their strengths and limitations, are presented elsewhere [12]. For this study, the national data sets were merged across the six countries to ensure adequate sample sizes by occupation and sex. We included the two largest occupation groups, physicians (15% of the sample) and nursing and midwifery personnel (45%), for a total of 2630 individual observations. Information on payments and compensation were analysed drawing on questions about occupational earnings as well as whether any of six different additional benefits were received at the place of work where they were interviewed: meals allowance, housing allowance, transport allowance, paid vacations, health insurance and in-service training accessed in the previous 12 months. The benefits were recorded as having been received or not, regardless of (real or perceived) value. While other types of benefits have been identified in the literature as used by employers for addressing worker productivity and retention, these were the six main non-pecuniary benefits covered in the questionnaire and for which comparable information was available. Given the cross-sectional nature of the survey, the results do not take into account workers who may have left a given facility or the health sector altogether due to unsatisfactory compensation.

Multiple regression models were used to investigate whether male and female health workers enjoy the same access to non-pecuniary benefits, controlling for other factors. Eight dichotomous dependent variables were employed, each taking the value 1 if the worker reported receiving the benefit, or 0 otherwise. Six variables were used for each of the six aforementioned benefits, plus two more variables for, respectively, whether at least one benefit was received and for whether at least three benefits were received.

The probability of each indicator (y_i_) taking the value 1 was investigated using a Probit regression of the following form:

$$\mathrm{Pr}\left[y_{i}=1|x\right]=\Phi \left({x_{i}}^{\prime }\beta \right)$$

where Φ denotes the standard normal cumulative distribution function, x_i _a vector of exogenous covariates and β the vector of associated coefficients.

The analysis considered a series of covariates considered likely to independently influence the outcome of interest. They included the sex of the worker, as well as the country context and other individual and facility-specific characteristics. Other individual characteristics included self-reported financial earnings and number of years of employment at the present facility. Facility-specific variables comprised the facility type (hospital/other), operating authority (government/other) and geographical location (urban/rural). Interaction variables were used to control for simultaneous influences across covariates. The analysis was done using the Stata statistical software package [14].

## Results

### Descriptives

**Figure 1:**
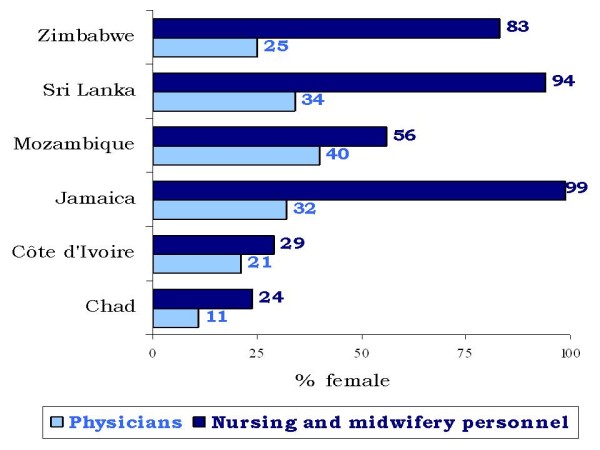
**Sex distribution of the facility-based health workforce, by country**. Source: Assessment of Human Resources for Health, 2002-2004 (n = 2630, unweighted survey data) [12].

Among the six countries under observation, the medical workforce is found to be predominantly male. Women make up only 31% of all surveyed physicians, ranging from 40% in Mozambique to 11% in Chad (Figure 1) [12]. Conversely, the nursing and midwifery workforce is mostly female: 75% of nurses and 98% of midwives are women. Here greater cross-national variations are observed, with the figures ranging from over 90% of nurses and midwives being women in Jamaica and Sri Lanka, to less than 30% in Chad and Côte d'Ivoire.

Table 1 presents descriptive statistics for facility-based staff receiving selected non-pecuniary benefits. Overall, the two most often received benefits are health insurance and access to in-service training, while meals allowance is least offered. However, wide variations can be found across countries and by sex. In Jamaica, for instance, women - who dominate the health workforce numerically - are found to generally receive more benefits compared to men. The opposite picture emerges for Chad, where most benefits (except paid holidays) are offered more frequently to men, the strong majority of the core medical cadres (physicians, nurses and midwives). The figures are relatively comparable between men and women in Sri Lanka and Zimbabwe, except for in-service training which tends to be more accessible to male staff.

**Table 1:** Percent of facility-based physicians, nurses and midwives receiving selected non-pecuniary benefits, by country and sex

	Chad		Côte d'Ivoire		Jamaica		Mozambique		Sri Lanka		Zimbabwe	
Benefit	Men	Women	Men	Women	Men	Women	Men	Women	Men	Women	Men	Women
Meals allowance	1%	0%	16%	26%	12%	15%	16%	18%	4%	2%	3%	1%
Housing allowance	24	19	11	5	6	2	18	20	2	2	91	92
Transport allowance	25	17	15	25	32	54	18	18	5	3	89	93
Paid vacations	72	77	18	20	36	80	22	25	10	10	84	92
Health insurance	48	38	10	13	18	43	26	32	64	73	11	17
In-service training	63	42	35	25	86	85	61	61	20	14	46	41
At least one benefit	93	89	58	61	94	97	78	78	75	78	97	99
Three or more benefits	40	27	12	14	30	64	23	24	5	5	86	90

### Results from the multiple regression analysis

The results from the regression analyses for nursing and midwifery personnel are reported in Table 2 and paint a complex picture. Across the six countries, after controlling for wages, years of experience and other variables, little gender difference is observed in terms of the likelihood of receiving any one, or several, of the identified non-monetary benefits (models 7 and 8, respectively). Looking at each benefit in turn, however, women are found somewhat more likely to receive transportation allowances (model 3) or health insurance (model 5) compared to their male counterparts. But they report significantly fewer opportunities for further professional training (model 6, *P *< 0.01).

**Table 2:** Results from the multiple regression models for the probability of nursing and midwifery personnel to receive non-pecuniary benefits, six countries

	(1)	(2)	(3)	(4)	(5)	(6)	(7)	(8)
Covariate	Meals	Housing	Transport	Paid vacation	Health insurance	In-service training	At least one benefit	Three or more benefits
**Worker's sex**:	-0.125	0.18	0.557**	-0.056	0.648***	-0.534***	0.325	0.089
Woman *(ref = man)*	[0.341]	[0.285]	[0.241]	[0.227]	[0.222]	[0.204]	[0.258]	[0.230]
**Worker's years of experience at facility**	-0.015	0	0.001	-0.014	0.018*	0.006	0.025*	0.002
	[0.016]	[0.013]	[0.012]	[0.012]	[0.011]	[0.010]	[0.015]	[0.011]
**Facility location**:	-0.419	0.221	0.069	-0.545***	0.354***	0.115	0.535***	-0.176
Rural *(ref = urban)*	[0.256]	[0.155]	[0.139]	[0.105]	[0.089]	[0.081]	[0.118]	[0.110]
**Facility type**:	0.878***	0.34	0.456**	-0.201	0.409**	-0.821***	0.112	0.078
Hospital *(ref = other)*	[0.300]	[0.252]	[0.215]	[0.196]	[0.195]	[0.177]	[0.219]	[0.198]
**Facility management**: Private *(ref = public)*	-0.174	0.58	-0.082	-0.834***	-0.075	-0.976***	-0.940***	-0.455
	[0.451]	[0.424]	[0.294]	[0.277]	[0.293]	[0.243]	[0.253]	[0.313]
Interaction sex*experience:	-0.007	0.001	-0.008	0.02	-0.016	0.001	-0.026	0.006
Woman*Years	[0.019]	[0.016]	[0.014]	[0.013]	[0.012]	[0.011]	[0.016]	[0.013]
Interaction sex*facility type:	0.112	-0.211	-0.600**	-0.012	-0.396*	0.633***	0.056	-0.222
Woman*Hospital	[0.372]	[0.306]	[0.258]	[0.238]	[0.228]	[0.213]	[0.275]	[0.241]
Interaction sex*facility management:	1.768***	0.116	1.213***	1.403***	-1.017***	0.507*	-0.058	1.052***
Woman*Private	[0.469]	[0.453]	[0.335]	[0.302]	[0.310]	[0.270]	[0.272]	[0.338]

*P < 0.1 **P < 0.05 ***P < 0.01 *ref *= reference category

Note: Additional variables for workers' country of residence and occupational earnings were included in the model, with generally highly statistically significant differences observed (results not presented, due in part to differences in currency scales across countries).

Hospitals are more likely to offer to their nursing and midwifery staff meals allowance, transport allowance and health insurance, but less often access to in-service training compared to other types of health facilities (e.g. health centres, maternity centres, health clinics, mobile clinics). Curiously, all else being equal, female hospital staff appear more likely to receive in-service training, and less likely to receive transport allowance or health insurance, compared to their male counterparts.

Private (non-government) health facilities tend to be less generous when it comes to staff benefits, less likely to offer paid vacations and access to in-service training than government-operated facilities. As demonstrated by the significant coefficients for the interaction term between facility management and health workers' sex, female nurses and midwives in private facilities tend to receive health insurance less often, whereas males receive relatively fewer paid holidays and trainings.

Among other potential confounding factors, years of work experience at the facility does not appear to have an independent influence on the probability of a nurse or midwife receiving a particular benefit, except health insurance.

The relevant results for physicians are reported in Table 3. Female physicians are found more likely to receive meals allowance, transportation allowance and paid vacations compared to their male counterparts. On the other hand, while, in general, hospitals are more generous with offering benefits to their medical staff, compared to men employed in hospitals, women are significantly less often in positions where they receive more benefits--including, specifically, meals, housing and transport allowances, as well as paid vacations.

**Table 3:** Results from the multiple regression models for the probability of physicians to receive non-pecuniary benefits, six countries

	(1)	(2)	(3)	(4)	(5)	(6)	(7)	(8)
Covariate	Meals	Housing	Transport	Paid vacation	Health insurance	In-service training	At least one benefit	Three or more benefits
**Worker's sex**:	1.869***	0.898	1.167**	0.907**	-0.002	-0.205	0.227	1.283***
Woman *(ref = man)*	[0.550]	[0.550]	[0.464]	[0.414]	[0.420]	[0.529]	[0.511]	[0.454]
**Worker's years of experience at facility**	-0.005	0.017	0.013	0.004	-0.006	0.006	0.015	-0.004
	[0.019]	[0.017]	[0.015]	[0.013]	[0.013]	[0.012]	[0.012]	[0.016]
**Facility location**:	-0.043	-0.022	-0.383*	-0.640***	0.689***	0.052	0.421**	-0.362*
Rural *(ref = urban)*	[0.279]	[0.260]	[0.200]	[0.176]	[0.149]	[0.132]	[0.165]	[0.211]
**Facility type**:	1.201***	1.234***	1.069***	0.809***	-0.075	0.141	0.336	1.203***
Hospital (*ref = other)*	[0.358]	[0.353]	[0.322]	[0.273]	[0.256]	[0.257]	[0.256]	[0.313]
**Facility management**:	1.466***	0.696***	0.157	0.084	-1.046***	-0.247	-1.218***	0.712***
Private *(ref = public)*	[0.262]	[0.263]	[0.240]	[0.198]	[0.187]	[0.200]	[0.184]	[0.219]
Interaction sex*experience:	-0.067	-0.013	-0.029	-0.031	-0.04	-0.009	-0.090***	0.007
Woman*Years	[0.047]	[0.036]	[0.033]	[0.030]	[0.029]	[0.035]	[0.033]	[0.033]
Interaction sex*facility type:	-1.543***	-0.937*	-1.003**	-0.715*	0.156	0.23	0.155	-1.385***
Woman*Hospital	[0.511]	[0.524]	[0.447]	[0.397]	[0.397]	[0.523]	[0.485]	[0.435]
Interaction sex*facility management:	-0.27	0.346	0.098	0.276	0.571		0.369	-0.332
Woman*Private	[0.471]	[0.475]	[0.443]	[0.374]	[0.348]		[0.355]	[0.444]

*P < 0.1 **P < 0.05 ***P < 0.01 *ref *= reference category

Note: Additional variables for workers' country of residence and occupational earnings were included in the model, with generally highly statistically significant differences observed (results not presented, due in part to differences in currency scales across countries).

No gender differences exist with regards to how government or private facilities manage medical staff. The years of employment at a health facility do not affect the likelihood of a physician receiving benefits, all else being equal.

## Discussion and conclusions

Addressing gender equity in health workforce policy and practice remains an ongoing challenge, in part due to limited interest among national and international HRH stakeholders, in part due to a deficient evidence base to inform decision making, especially in low- and middle-income countries. This paper sought to expand the existing knowledge base, and to encourage researchers to mainstream gender issues in future health workforce analyses. For example, among the 262 articles published in the *Human Resources for Health *journal between its inception in April 2003 and September 2011, only 89 (34%) paid any mention of the word 'gender' in the text, a mere 14 (5%) paid mention in the abstract, and just one (0.4%) [15] in the title. And this despite gender imbalances having been identified as one of the four key dimensions to understanding health workforce imbalances for policy decision making [16]. Monitoring the gender aspect of the health workforce requires better measures of men and women in the health workforce, to help identify and prioritize evidence-based gender-sensitive HRH planning and management interventions [1]. The need to draw attention to the consequences and costs of failing to address both women's health needs and their contribution to the health of societies is globally recognized [17]. Accumulation and validation of gender-based HRH research and analysis will help ensure that the right questions are being asked and provide greater clarity when making decisions.

The central point of this analysis was gender differences in compensation of health personnel, focusing on access to non-monetary benefits, a previously neglected area of research. From a theoretical perspective, like all work settings, health facilities might find it beneficial to offer non-monetary benefits. Non-pecuniary benefits may represent value added for employees, making health facilities that offer these better able to attract and retain staff. To improve rural retention of health workers, the World Health Organization's new global policy guidelines recommend the use of fiscally sustainable incentives, such as grants for housing or paid vacations, to offset workers' perceived opportunity costs of working in rural areas [18]. However the guidelines acknowledge there is inconclusive evidence about the extent to which gender is associated with practising in rural areas, and do not recommend any gender-specific interventions given the lack of evidence on which incentives may be more amenable to female or male health workers.

Our empirical analysis of facility-based survey data in six countries, conducted through a gender lens, revealed differing patterns in employment conditions. While the analysis did not uncover any consistent pattern of gender imbalance, some important differences were revealed, and this despite the lack of any explicit gender-based policy. Notably, female nursing and midwifery personnel (who represent the majority in the sample) are found significantly less likely than their male counterparts to access in-service training, identified not only as an incentive to attract and retain workers but also essential for strengthening human capital and workforce quality. It is possible, at least outside of hospital settings, that such a result may be a reflection of subtle forms of gender bias, whereby female service providers' contributions are less valued and their opportunities for avenues for personal and professional growth beyond the basic health care tasks with which they were originally charged remain more limited [3]. Such findings highlight the critical need for additional context-specific research using sex-disaggregated data in order to better understand women's and men's contributions to health systems functioning and status in the workforce, within and across occupations.

Given its exploratory nature, this analysis was subject to certain limitations. It remains uncertain whether any of the findings can be considered generalizable, given the diverse social, economic and health system environments across the six countries under observation, as well as certain technical constraints--including varying national survey sample sizes and coverage [12], plus lack of information on other potential benefits, workers' choices and perceptions of the value of different benefits, and alternative sources of employer-provided benefits. The present results were perhaps limited in terms of their application to inform HRH policy and practice in a given context, especially in light of the very different histories, cultures and practice regulations, across health occupations among countries. However, it is hoped the approach will stimulate further data and research generation (quantitative and qualitative) to better understand health labour market dynamics, and with particular attention to gender dimensions.

## Competing interests

The authors declare that they have no competing interests.

## Authors' contributions

NG conceptualised the study design and contributed in the development of the survey instruments. MA conducted database management and statistical software programming. Both authors contributed to writing and interpretation of findings, and read and approved the final version.
